# Surgical results and prognostic factors following percutaneous full endoscopic posterior decompression for thoracic myelopathy caused by ossification of the ligamentum flavum

**DOI:** 10.1038/s41598-020-58198-x

**Published:** 2020-01-28

**Authors:** Xingchen Li, Bo An, Haoran Gao, Chengpei Zhou, Xiaobing Zhao, Haijun Ma, Bisheng Wang, Hejun Yang, Honggang Zhou, Xinjun Guo, Huimin Zhu, Jixian Qian

**Affiliations:** 1 Department of Spinal Surgery, Third Hospital of Henan Province, Zhengzhou, Henan Province P.R. China; 2 Department of Orthopedics, Tangdu Hospital affiliated with Air Force Medical University, Xi’an, Shanxi Province P.R. China; 3 Department of Spinal Surgery, Armed Police Corps Hospital of Henan Province, Zhengzhou, Henan Province P.R. China

**Keywords:** Spinal cord diseases, Neurosurgery, Risk factors

## Abstract

Minimally invasive surgery (MIS) has shown satisfactory surgical results for the treatment of thoracic myelopathy (TM) caused by ossification of the ligamentum flavum (OLF). This study investigated the prognostic factors following MIS and was based on the retrospective analysis of OLF patients who underwent percutaneous full endoscopic posterior decompression (PEPD). Thirty single-segment OLF patients with an average age of 60.4 years were treated with PEPD under local anaesthesia. Clinical data were collected from the medical and operative records. The surgical results were assessed by the recovery rate (RR) calculated from the modified Japanese Orthopaedic Association (mJOA) score. Correlations between the RR and various factors were analysed. Patients’ neurological status improved from a preoperative mJOA score of 6.0 ± 1.3 to a postoperative mJOA score of 8.5 ± 2.0 (*P* < 0.001) at an average follow-up of 21.3 months. The average RR was 53.8%. Dural tears in two patients (6.7%, 2/30) were the only observed complications. Multiple linear regression analysis showed that a longer duration of preoperative symptoms and the presence of a high intramedullary signal on T2-weighted MRI (T2HIS) were significantly associated with poor surgical results. PEPD is feasible for the treatment of TM patients with a particular type of OLF. Patients without T2HIS could achieve a good recovery if they received PEPD early.

## Introduction

Thoracic myelopathy (TM) is less common than cervical myelopathy and lumbar spinal stenosis^1^, and TM is mainly caused by ossification of the ligamentum flavum (OLF) in East Asian countries, such as Japan, Korea, and China^2^. As the number of reported cases has increased, OLF has been studied not only in East Asia but also worldwide^3–5^. Although much of its pathophysiology has been determined, the exact pathogenetic mechanism and the epidemiology of OLF remain poorly understood^6,7^. Therefore, making an appropriate and timely therapeutic decision for the treatment of OLF may be hindered by the paucity of knowledge. TM caused by OLF remains a challenge for spine surgeons.

OLF generally requires posterior surgical decompression due to its progressive nature and poor response to conservative therapy^8,9^. Decompression procedures include traditional open surgeries, such as laminectomy with or without posterior fusion^10,11^, and minimally invasive surgery (MIS), such as microendoscopic decompression^12,13^ and percutaneous endoscopic decompression^14–17^. However, the prognostic guidelines are still unclear, and the surgical results vary widely despite complete decompression^3,18^.

To the best of our knowledge, all the published studies on prognostic factors in OLF patients have been based on traditional open surgery. However, MIS has been advantageous in treating OLF with satisfactory surgical results^12,15–17^. This study investigated the prognostic factors following MIS and was based on the retrospective analysis of OLF patients who underwent percutaneous full endoscopic posterior decompression (PEPD).

## Materials and Methods

The Ethics Committee of Tangdu Hospital approved the study, and all patients signed informed consent. The methods described were performed in accordance with relevant guidelines and regulations. This was not a study commissioned or funded by any manufacturer.

### Patient population

Between April 2016 and January 2018, thirty consecutive patients with TM caused by single-segment OLF underwent PEPD under local anaesthesia using the endoscopic system (iLESSYS^®^, Joimax^®^ GmbH, Karlsruhe, Germany). The indications for surgery were progressive muscle weakness in the lower extremities, gait disturbance, dorsal pain, and sphincter dysfunction. Neurological examinations and preoperative MRI/CT were performed to determine the location of OLF and the target area for decompression. OLF was classified into lateral, extended, enlarged, fusion, and tuberous types based on axial CT as well as round and beak types based on sagittal MRI^19,20^. Fusion and tuberous types of OLF were excluded based on the surgical difficulties in endoscopic decompression^15^. In addition, patients with ventral compression (thoracic disc herniation, ossification of the posterior longitudinal ligament), concomitant cervical or lumbar lesions and severe cardiopulmonary disease were also excluded. The clinical characteristics were recorded, including sex, age, the duration of preoperative symptoms, a history of smoking and comorbidities (diabetes mellitus or hypertension). The presence of a high intramedullary signal on T2-weighted MRI (T2HIS) was recorded. The location of the lesions was classified as upper (T1-T4), middle (T5-T8), and lower (T9-T12) on the basis of the thoracic spine level. The presence of intraoperative dural adhesion/dural ossification (DA/DO), operation time and estimated blood loss (EBL) were also recorded.

### Clinical and radiologic assessments

**Figure 1:**
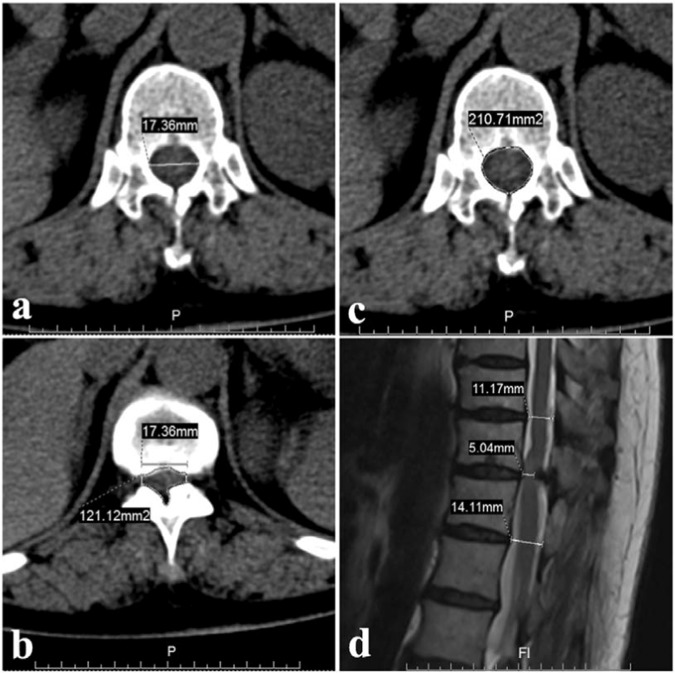
The measurement of the CSA and APD on axial CT and sagittal MRI (case 21). (**a**) The widest distance between two pedicles as viewed on a CT scan was measured as the transverse spinal canal diameter, equal to the transverse spinal canal diameter at the maximally compressed CT scan. (**b**) A vertical line extending through the endpoints of the transverse diameter determined the boundary of the spinal canal and was used to measure the compressed CSA. (**c**) The normal CSA was measured on the pedicle section of the same vertebrae. (**d**) The APD was measured at the compressed level as well as at two normal levels above and below the compressed level.

All patients were followed for at least one year. Pre- and postoperative neurological statuses were evaluated using the modified Japanese Orthopaedic Association (mJOA) score (Table 1). The recovery rate (RR) = (postoperative mJOA-preoperative mJOA)/(11-preoperative mJOA) × 100%^21^. According to the RR, the surgical results were divided into good (50–100%), fair (25–49%), unchanged (0–24%), or deteriorated (<0%)^22^. The cross-section area (CSA) and the anteroposterior diameter (APD) of the spinal canal (Fig. 1) were measured on axial CT and sagittal T2-weighted MRI using the Picture Archiving and Communication Systems (PACS). The CSA was measured as follows^23^: The widest distance between two pedicles as viewed on a CT scan was measured as the transverse spinal canal diameter (Fig. 1a), equal to the transverse spinal canal diameter at the maximally compressed CT scan. A vertical line extending through the endpoints of the transverse diameter determined the boundary of the spinal canal and was used to measure the compressed CSA (Fig. 1b). The normal CSA was measured on the pedicle section of the same vertebrae (Fig. 1c). The ratio of CSA = (the compressed CSA)/(the normal CSA) × 100%. The APD was measured at the compressed level and at two normal levels above and below the compressed level (Fig. 1d)^6^. The average value of the APD just above and below the affected segment was the normal APD. The ratio of APD = (the compressed APD)/(the normal APD) × 100%.

**Table 1:** Summary of the mJOA scoring system for the assessment of thoracic myelopathy.

Neurological status	Score
Lower-limb motor dysfunction	
No dysfunction	4
Lack of stability and smooth reciprocation of gait	3
Able to walk on flat floor with walking aid	2
Able to walk up/downstairs with handrail	1
Unable to walk	0
Lower-limb sensory deficit	
No deficit	2
Mild sensory deficit	1
Severe sensory loss or pain	0
Trunk sensory deficit	
No deficit	2
Mild sensory deficit	1
Severe sensory loss or pain	0
Sphincter dysfunction	
No dysfunction	3
Minor difficulty with micturition	2
Marked difficulty with micturition	1
Unable to void	0

### Surgical techniques

**Figure 2:**
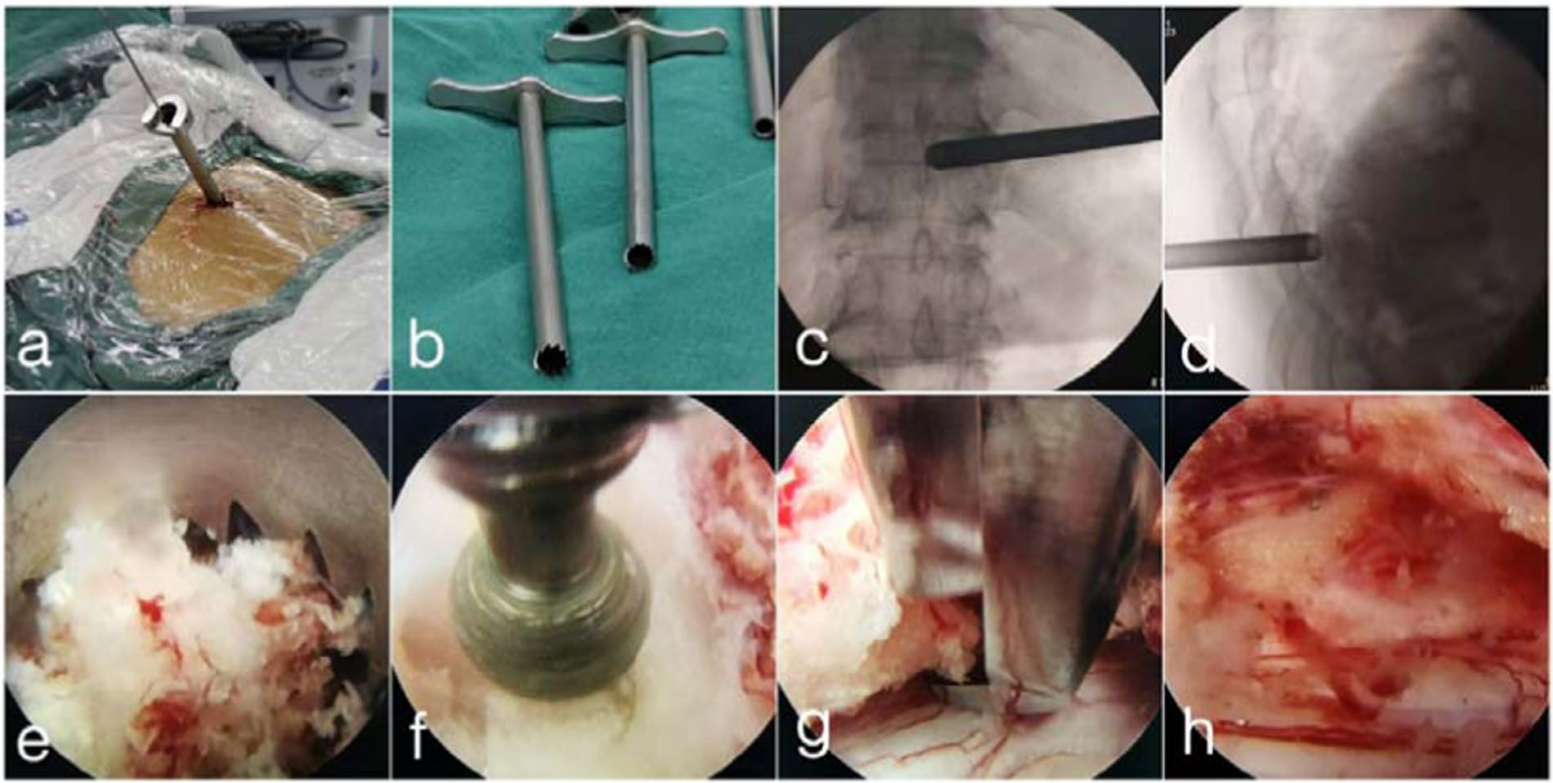
Intraoperative views of PEPD (case 21). (**a**,**b**) A specially designed bevelled working cannula was placed, and a specially designed circular saw was placed through the cannula. (**c**,**d**) Fluoroscopic views of the circular saw. (**e**) Laminotomy was achieved via the circular saw under the view of the endoscope. (**f**) Diamond abrasor was used to grind the contralateral ossified LF into a thin and translucent shape. (**g**) Endo-Kerrison punch was used to remove the remnant ossified LF. (**h**) The dural sac was exposed, and pulsation of the dural sac improved.

The patient was carefully arranged in the prone position. According to anaesthesiologists, dexmedetomidine (0.2–0.7 μg/kg/min) and sufentanil (0.1 μg/kg) were used to alleviate pain and maintain the sober situation. Patient feedback could be received promptly during the operation. The entry point in the skin was 5–6 cm from the midline. After the infiltration of local anaesthetics (0.5% lidocaine), an 18-gauge spinal needle was introduced under fluoroscopic guidance to the lamina on the root of the spinous process. Then, an approximately 10-mm skin incision was made, and the dilation catheter was inserted in sequence. A specially designed 8.5-mm diameter bevelled working cannula was placed, and a specially designed 7.5-mm diameter circular saw was placed through the cannula (Fig. 2a–d). Finally, the endoscope was placed through a circular saw, and laminotomy was achieved under the view of the endoscope (Fig. 2e). The unossified ligamentum flavum (LF) and soft tissue were removed by forceps and radiofrequency. Diamond abrasors were used to grind the contralateral ossified LF into a thin and translucent shape (Fig. 2f), and then Endo-Kerrison punches were used to remove the remnant ossified LF (Fig. 2g). The ipsilateral lesion was treated in the same manner. The whole procedure utilized the technique of “over-the-top” decompression^15^. Finally, the dural sac was exposed, and pulsation of the dural sac improved (Fig. 2h). Notably, tight DA/DO should be maintained but completely isolated from the surrounding LF to avoid a dural tear^15^. After complete decompression, the cannula was removed, and the incision was closed without the use of a suction drain.

### Statistical analysis

The data were analysed using Statistical Package for the Social Sciences (SPSS) software (version 18; IBM Corp., Armonk, NY). Student’s t-tests and one-way analysis of variance were used to compare the statistical significance of the association for continuous data. Pearson’s rank correlation coefficients were used to test the correlations between various factors and the RR. Multiple linear regression analysis was conducted to determine the quantitative variables that best correlated with the surgical results. A *P* value less than 0.05 was considered statistically significant. Qualitative data were converted to numbers for quantitation as follows^24^: (1) Sex: male, 1; female, 2. (2) Location of lesion: upper, 1; middle, 2; lower 3. (3) CT classification: lateral, 1; extended 2; enlarged 3. (4) MRI classification: round 1; beak 2. (5) Diabetes mellitus (DM): absent, 1; present 2. (6) Hypertension: absent, 1; present 2. (7) History of smoking: absent, 1; present 2. (8) T2HIS: absent, 1; present 2. (9) Intraoperative DA/DO: absent, 1; present 2.

## Results

### Clinical characteristics

This study included 17 male patients and 13 female patients with an average age of 60.4 (44–84) years. The patients’ symptoms included weakness and paraesthesia in the lower extremities, gait instability, claudication and sphincter dysfunction. Hyperreflexia occurred in all patients. The average duration of preoperative symptoms was 17.4 (0.4–40) months. Three patients (10.0%) were diagnosed with DM, 13 patients (43.3%) with hypertension, and 7 patients (23.3%) with a history of smoking before surgery.

### Radiographical findings

**Figure 3:**
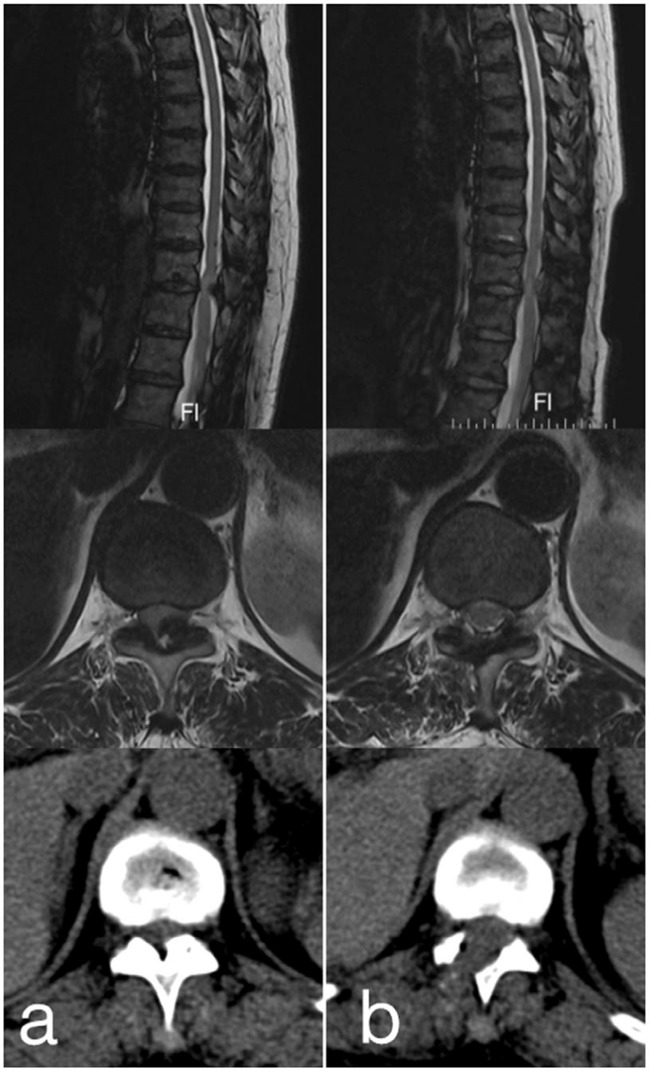
Pre- and postoperative images of PEPD (case 21). (**a**) Sagittal MRI, axial MRI and CT revealed the OLF at T11/12 and the compressed spinal cord. (**b**) Satisfactory decompression was completed with a dome-shaped laminotomy through limited laminectomy and flavectomy.

There were 24 lesions (80%) located at the lower thoracic spine, 4 lesions (13.3%) at the middle, and 2 lesions (6.7%) at the upper. According to axial CT images, 3 cases (10.0%) were classified as lateral type, 3 (10.0%) cases were extended type, and 24 (80.0%) cases were enlarged type. According to the sagittal MRI images, 20 cases (66.7%) were classified as round type, and 10 (33.3%) were classified as beak type. T2HIS was observed in 16 cases (53.3%). The mean APD and CSA of the normal thoracic canal were 11.6 (7.7–17.4) mm and 167.0 (110.1–296.0) mm^2^, and the mean APD and CSA of the compressed level were 5.4 (3.3–8.9) mm and 109.1 (66.3–195.9) mm^2^. The mean ratios of the APD and CSA were 47.3% and 66.2%, respectively. According to the pre- and postoperative CT and MR images, decompression was completed successfully by PEPD with a dome-shaped laminotomy through limited laminectomy and flavectomy (Fig. 3).

### Surgical outcome

In general, patients’ neurological status improved from a preoperative mJOA score of 6.0 ± 1.3 to a postoperative mJOA score of 8.5 ± 2.0 points (*P* < 0.001) at an average follow-up time of 21.3 months, yielding an average RR of 53.8%. According to the RR, 16 (53.3%) cases were classified as good, 7 (23.3%) cases were fair, 7 (23.3%) cases were unchanged, and 0 cases were deteriorated. The mean operation time was 167.0 (100–240) minutes. The mean EBL was 36.2 (25.0–50.0) ml. During PEPD, we found DA/DO in 11 (36.7%) patients, in whom 2 patients (cases 8 and 18) experienced dural tears, yielding an overall incidence of 6.7% (2/30). However, the two patients did not receive repair or indwelled drainage. They healed after staying in a prone position for one week with a pressure dressing. Neither cerebrospinal fluid cysts nor incision dehiscence occurred during their follow-up. No neurological deficits or other complications occurred in this study.

### Relationship of the RR to various factors

Univariate analysis revealed that a poor RR was significantly related to a longer duration of symptoms, a lower preoperative mJOA score, a lower ratio of APD, a lower ratio of CSA, the presence of DM, the presence of T2HIS, and the presence of intraoperative DA/DO (Table 2).

**Table 2:** Relationships between the recovery rate and various factors.

Factor		N	RR (%)	*P* value
Sex	Male	17	45.4 ± 30.3	0.102
	Female	13	64.7 ± 31.6	
Location of lesion	Upper	2	70.0 ± 42.4	0.191
	Middle	4	77.2 ± 29.7	
	Lower	24	48.5 ± 30.6	
CT classification	Lateral	3	46.0 ± 27.6	0.305
	Extended	3	80.6 ± 17.3	
	Enlarged	24	51.4 ± 32.8	
MRI classification	Round	20	50.6 ± 31.8	0.447
	Beak	10	60.1 ± 32.7	
DM	No	27	56.7 ± 32.2	0.005
	Yes	3	27.5 ± 9.0	
Hypertension	No	17	55.3 ± 36.5	0.766
	Yes	13	51.7 ± 25.8	
History of smoking	No	23	55.7 ± 34.3	0.562
	Yes	7	47.5 ± 22.7	
T2HIS	No	14	74.7 ± 24.7	0.000
	Yes	16	35.4 ± 25.7	
Intraoperative DA/DO	No	19	67.3 ± 29.0	0.001
	Yes	11	30.4 ± 22.0	
Age at surgery			R = −0.301	0.106
Duration of symptoms			R = −0.729	0.000
Preoperative mJOA score			R = 0.455	0.011
Operation time			R = 0.219	0.245
EBL			R = 0.145	0.443
Ratio of APD			R = 0.661	0.000
Ratio of CSA			R = 0.553	0.002

^a^CT denotes computed tomography; ^b^MRI denotes magnetic resonance imaging; ^c^DM denotes diabetes mellitus; ^d^T2HIS denotes high intramedullary signal on T2-weighted MRI; ^e^DA denotes dural adhesion; ^f^DO denotes dural ossification; ^g^mJOA denotes modified Japanese Orthopaedic Association; ^h^EBL denotes estimated blood loss; ^i^APD denotes anteroposterior diameter; ^j^CSA denotes cross-section area.

### Prognostic factors related to the surgical results

Multiple linear regression analysis showed that a longer duration of preoperative symptoms and the presence of T2HIS were significantly associated with a poor RR (Table 3).

**Table 3:** Independent factors associated with recovery rate.

Factor	Partial regression coefficient (B)	Standardized partial regression coefficient (Beta)	*P* value
	100.301 (constant)		
Sex	2.117	0.034	0.853
Age at surgery	−0.272	−0.085	0.521
History of smoking	−2.926	−0.040	0.822
DM	−25.975	−0.249	0.166
Hypertension	15.421	0.244	0.082
Duration of symptoms	−1.114	−0.430	0.013
Preoperative mJOA score	3.130	0.132	0.435
Location of lesion	−8.789	−0.161	0.246
CT classification	3.330	0.068	0.745
MRI classification	−3.134	−0.047	0.719
T2HIS	−27.950	−0.445	0.012
Ratio of APD	0.267	0.077	0.654
Ratio of CSA	0.017	0.007	0.978
Operation time	0.076	0.101	0.507
EBS	0.407	0.102	0.437
Intraoperative DA/DO	−1.184	−0.018	0.923

^a^DM denotes diabetes mellitus; ^b^mJOA denotes modified Japanese Orthopaedic Association; ^c^CT denotes computed tomography; ^d^MRI denotes magnetic resonance imaging; ^e^T2HIS denotes high intramedullary signal on T2-weighted MRI; ^f^APD denotes anteroposterior diameter; ^g^CSA denotes cross-section area; ^h^EBL denotes estimated blood loss; ^i^DA denotes dural adhesion; ^j^DO denotes dural ossification.

## Discussion

Percutaneous endoscopic surgery, which is the most minimally invasive spinal surgery, can achieve decompression of spinal stenosis based on improvements in equipment and optical technology^25^. To the best of our knowledge, this is the first study about the prognostic factors of OLF treated by MIS and the largest study about the surgical results of PEPD for the treatment of OLF. We found that PEPD was feasible for the treatment of thoracic OLF, and OLF patients without T2HIS could achieve a good recovery if they received PEPD early.

OLF mostly occurs in the lower thoracic spine and mostly affects male adults aged 40 to 60 years^7,24,26–28^. We found similar results, and our data showed that 56.7% (17/30) of the patients were male, 53.3% (16/30) were less than 60 years of age, and 80% (24/30) had decompression at the lower thoracic spine. In this study, patients’ neurological status improved significantly after PEPD, and the average RR was 53.8%, which is comparable to the 16–58.7% reported by several studies^6,24,27,29,30^.

Patients may suffer kyphosis after traditional open laminectomy because of the removal of bone tissue and destruction of the posterior tension band^18,31,32^. Posterior instrument fusion is recommended but still controversial. Yu *et al*. found that the overall mean increase in kyphosis was only 2.9° at a minimum of 1 year after surgery in 49 OLF patients, and no patient required additional surgery due to spinal deformity^24^. Aizawa *et al*. suggested that there was no relationship between surgical outcomes and an increased kyphotic angle after laminectomy^31^. In addition, thoracic kyphosis might be influenced by changes in back extensor strength in particular, and providing strong, natural extrinsic support for the spine seems to be important for decreasing the incidence of spinal deformities^33^. Percutaneous endoscopic surgeries minimize damage to the paraspinal muscles, facet joints, lamina and posterior ligamentous complexes^15^. We believe that OLF patients who undergo PEPD without fusion may have a much lower risk of kyphosis, and we will continue to conduct a control study to verify this point.

There is great interest in surgical techniques that can minimize complications. Recently, a meta-analysis showed a moderately high rate of perioperative complications after laminectomy for OLF, and the incidence of dural tears, cerebrospinal fluid (CSF) leaks, infections, and early neurological deficits were 18.4%, 12.1%, 5.8%, and 5.7%, respectively^34^. It is promising that the incidence of complications reported in MIS has been much lower^12,15^. The incidence of dural tears was only 6.7% (2/30) in this study. Since the diameter of punches and forceps used in PEPD was very small and the endoscopic visualization of anatomical structures in a liquid environment was very clear, endoscopic manipulation was so gentle that dural tears could be avoided as much as possible. However, tight DA/DO should remain *in situ* as a floating adherent fragment^12,15^.

Several studies have reported that some factors might affect the surgical results of OLF, including the duration of preoperative symptoms, preoperative neurological status, intramedullary signal changes in T2WI, age, sex, type of OLF, and dural tears^3,7,20,27,30,35–37^. However, it is still uncertain whether these factors are predictive of the surgical results following MIS.

The duration of preoperative symptoms was confirmed to be significantly correlated with the surgical results in our study, which is consistent with many studies^3,19,29,35,38,39^. We believe that the exacerbation of preoperative neurological status over a long time is mostly irreversible. Thus, earlier surgical intervention might result in better clinical outcomes, regardless of traditional laminectomy or MIS.

The presence of T2HIS was confirmed to be significantly correlated with surgical results in our study, which is similar to some studies^7,30^. Intramedullary signal changes in the spinal cord occur during pathologic changes, such as the loss of nerve cells, oedema, gliosis, demyelination, and Wallerian degeneration^40,41^. We believe that patients with intramedullary signal changes have irreversible spinal cord changes. As the symptoms are subjective, objective findings, such as radiologic examinations, are important for evaluating the factors that affect surgical results^30^.

The severity of myelopathy before surgery, which is mostly indicated by the preoperative mJOA score, was shown to be the most important predictor of surgical results in many studies^3,24,36,38,41–43^. However, few studies found a significant correlation between the preoperative mJOA score and RR according to multiple regression analysis^3,36,41^, and one study even showed no significant correlation between them in the univariate analysis^1^. Of note, we did not find a significant correlation between the preoperative mJOA score and RR in multiple regression analysis, similar to some studies^24,29,38^. A lower preoperative mJOA score may result in worse recovery, but it is not a predictor of the surgical results following PEPD.

The morphology of OLF might affect the surgical results but is still controversial. Kuh *et al*. suggested that the beak type of OLF with high intramedullary signal changes might be a poor prognostic factor^20^. However, Kang *et al*. found that patients with beak type OLF could achieve satisfactory RRs and considered that localized compression of the spinal cord could be corrected better than diffuse compression of a round type OLF^42^. Previous studies showed that the RR was significantly better in non-fused compression patients than in fused patients according to axial CT images^6^. However, Li *et al*. found that the axial CT configurations (unilateral, bilateral or bridged) were not significantly related to the surgical results^41^. Ando *et al*. also found that there were no statistically significant differences in the RR among the five OLF types (lateral, extended, enlarged, fused, and tuberous)^27^, which was similar to our results.

As the degree of compression increases, the stress distribution on the spinal cord increases. Our data showed that the ratio of APD and CSA had a negative influence on recovery in the univariate analysis, which is consistent with some studies^24,29^. However, we did not find any statistical correlation between the RR and the ratio of APD/CSA in multiple regression analysis. According to the surgical criteria of PEPD^15^, we excluded the fusion and tuberous types of OLF, which both tend to have poor surgical results because of the severe compression to the spinal cord and the low APD/CSA ratio^6,19,24^. The insufficient types of OLF in this study might make it difficult for us to draw significant correlations between the RR and the APD/CSA ratio, as well as between the RR and the types of OLF.

OLF located in the middle thoracic spine may be a predictor of poor outcome^24^. The middle thoracic spine, as the watershed area of the spinal circulation, has the narrowest canal and the maximal kyphotic angle. Thus, the spinal cord in the middle thoracic spine had insufficient compensatory space during posterior decompression, and there is a much greater risk of iatrogenic injury and ischaemic damage^44^. However, we did not find a significant correlation between the location of the lesion and RR. In our study, all four patients (cases 8, 17, 23, 28) with OLF located in the middle thoracic spine achieved satisfactory surgical results (good, fair, good, and good). PEPD showed features including small trauma, high safety (local anaesthesia), and fine and accurate manipulation^15^. It is advantageous to use PEPD to treat OLF, especially to treat OLF located in the middle thoracic spine.

Thoracic OLF is commonly associated with other spinal disorders, such as disc herniation and ossification of the posterior longitudinal ligament (OPLL). Vascular injury is more likely to occur in anterior lesions such as OPLL, which could result in more severe myelopathy and worse recovery regardless of the less severe stenosis^28,44^. Therefore, we excluded patients with coexisting spinal conditions because their therapeutic decisions were more complicated and the surgical results were more unpredictable^6,41^.

The main limitation of this study was the lack of a control group which considered as the gold standard, such as the laminectomy. If the control group was established, the significance of this research would be greatly strengthened. We had started to conduct a retrospective case-control study in which laminectomy is used as a control group, and relevant data were being collected and analyzed. In addition, it was a retrospective study with a relatively small number of patients, and only included single-segment and incomplete types of OLF patients, which could have an impact on the adequacy of our results and could have weakened the statistical significance. Nonetheless, we believe that our results may provide preliminary data in support of the guidelines for PEPD to treat OLF. We will continue to refine this minimally invasive approach.

## Conclusions

PEPD, as the most minimally invasive spinal decompression surgery, is feasible for the treatment of TM patients with a particular type of OLF. A longer preoperative duration of symptoms and the presence of T2HIS were significantly associated with poor surgical results following PEPD. Patients without T2HIS could achieve a good recovery if they received PEPD early.
